# MCT4 inhibition attenuates inflammatory response to *Mycobacterium avium paratuberculosis* infection and restores intestinal epithelial integrity *in vitro*

**DOI:** 10.3389/fimmu.2025.1562100

**Published:** 2025-04-14

**Authors:** Ala’ Alhendi, Saleh A. Naser

**Affiliations:** Division of Molecular Microbiology, Burnett School of Biomedical Sciences, College of Medicine, University of Central Florida, Orlando, FL, United States

**Keywords:** MCT4, *Mycobacterium avium paratuberculosis* (MAP), TLR-2, Crohn’s disease (CD), SERPINE1, tight junction

## Abstract

**Introduction:**

*Mycobacterium avium paratuberculosis* (MAP) plays a significant role in Crohn’s disease (CD). Monocarboxylate transporter 4 (MCT4) is a proton-coupled symporter of lactate that facilitates the inflammatory shift in macrophages and increases their reliance on glycolysis. MCT4 is also involved in the negative regulation of intestinal epithelial barrier function.

**Methods:**

In this *in vitro* study, we examined the role of MCT4 in macrophages and its effect on intestinal epithelial homeostasis during MAP infection. We used cultured THP-1 macrophages infected with a clinical strain of MAP (UCF4) as well as intestinal cell lines, Caco-2 and HT-29. MCT4 was inhibited using α-cyano-4-hydroxycinnamic acid (CHCα).

**Results:**

Infection of THP-1 cells with MAP upregulated MCT4 expression (2 folds) and resulted in a significant increase in lactate export (1.3 folds), TNFα (13.8 folds), and IL-6 (1.3) via TLR2 activation. Consequently, intestinal damage markers were also upregulated, including MUC2 (2.5 folds), NOX-1 (2 folds), SERPINE1 (2.1 folds), IL-6 (1.6 folds), and CLDN2 (1.4 folds). Inhibition of MCT4 during MAP infection with CHCα significantly reduced TNF-α and IL-6 levels. This effect on macrophages restored baseline oxidative status and mucin production in HT-29 intestinal cells. Moreover, MCT4 inhibition in a MAP-infected THP-1-Caco-2 co-culture system restored IL-6 and SERPINE1 to normal levels and enhanced tight junction protein, TJP1 (ZO-1), expression.

**Conclusion:**

Collectively, this study revealed the significant role of MCT4 in CD pathophysiology during MAP infection and highlighted MCT4 as a potential therapeutic target for CD treatment.

## 1 Introduction

Crohn’s disease (CD) is a chronic inflammatory condition of the gastrointestinal tract that is characterized by a multifactorial etiology (1). These include genetic susceptibility, environmental stimuli, impaired gut microbiota, or a combination of these factors (2, 3). *Mycobacterium avium paratuberculosis* (MAP) is a suspected microbial agent responsible for CD inflammation in almost 50% of the patients. MAP has been isolated from the blood, intestinal tissues, and body fluids of CD patients (4–6). MAP survives in host macrophages by preventing the phagolysosomal degradation of microbes (5). It upregulates the release of inflammatory cytokines by macrophages, such as TNFα, IL-6, and IFNγ, which contribute to inflammation progression and intestinal epithelial dysfunction (7, 8).

Monocarboxylate transporter 4 (MCT4), encoded by the solute carrier 16 gene *SLC16A3*, is a bidirectional proton-coupled symporter of lactate (9). Among other MCTs, MCT4 is highly expressed by glycolytic cells, such as skeletal muscle cells, astrocytes, immune cells, chondrocytes, and hypoxic tissues (9). MCT4 has a lower affinity for pyruvate than lactate, ensuring that pyruvate is converted to lactate via an NAD^+^-regenerating reaction before being exported extracellularly (9, 10). NAD^+^ regeneration is essential for driving glycolytic flow in these tissues. MCT4 is the only MCT that is upregulated in response to *Mycobacterium tuberculosis* (*Mtb*) infection in mouse lungs, whereas other MCTs are downregulated or unresponsive (11). MCT4 is also upregulated by macrophages activated by TLR2 or TLR4 agonists to maintain increased glycolytic demand and, consequently, lactate production. This is crucial for sustaining the inflammatory response of macrophages and production of pro-inflammatory cytokines (12).

MCT4 expression is upregulated in the inflamed colonic mucosa of inflammatory bowel disease (IBD) patients. This finding was associated with increased blood lactate levels in the same patients. Moreover, MCT4 expression is positively correlated with the severity of intestinal inflammation, supporting MCT4 as a prognostic candidate for IBD inflammation and disease activity (13, 14). Ectopic expression of MCT4 in cultured intestinal cells compromises barrier function and upregulates inflammatory IL-6 release (14). Moreover, MCT4 overexpression in cultured intestinal cells upregulates inflammasome-mediated cell pyroptosis and cleavage of the inflammatory precursors IL-18 and pro-IL-1β (15). This aggravates intestinal inflammation via the ERK1/2 and NF-κB pathways, leading to NLRP3 inflammasome and caspase-1 activation (15).

Given the dual involvement of MCT4 in inflammatory activation of macrophages and intestinal barrier dysfunction, we investigated the role of MCT4 during MAP infection. The effect of MCT4 inhibition on intestinal cells and macrophages during microbial infection has not yet been explored. This also includes the consequences of this inhibition in restoring intestinal epithelial integrity and the ability of intestinal tissue to heal after injury. Using an *in vitro* model of MAP-infected macrophages co-cultured with intestinal epithelial cell lines, we identified the effects of MCT4 inhibition using α-cyano-4-hydroxycinnamic acid (CHCα) on inflammatory markers and intestinal epithelial integrity during MAP infection. Selective and non-selective small molecule inhibitors of MCT4 are currently in the drug discovery and preclinical experimentation phases and are yet to be used in clinical trials for cancer treatment (16–18). Here, we propose the use of MCT4 inhibition to alleviate intestinal epithelial damage in MAP-positive CD patients.

## 2 Materials and methods

### 2.1 Cell lines and culture conditions

THP-1 monocytes (ATCC TIB-202), Caco-2 intestinal cells (ATCC HTB-37), and HT-29 intestinal cells (ATCC HTB-38) were maintained and differentiated according to our previously described protocol (8). Differentiated THP-1 macrophages were either left as controls or infected with 1 × 10^7^ CFU/mL of the clinical MAP strain, UCF4 (isolated from CD patients), for 24 h before RNA extraction and supernatant collection.

### 2.2 RNA extraction, reverse transcription, and qRT-PCR

Cellular RNA was extracted from control and treated cells using the RNeasy^®^ Mini Kit (Qiagen, Hilden, Germany), following the manufacturer’s instructions. RNA (1,000 ng) was used in a reverse transcription reaction to generate cDNA using a high-capacity cDNA reverse transcription kit (Thermo Fisher Scientific, Waltham, MA, USA). cDNA was diluted 2:23 for THP-1, 1:24 for Caco-2, and 1:9 for HT-29 cells in nuclease-free water, and the real-time PCR reaction was performed as previously described using the QuantStudio™ Real-Time PCR instrument (Applied Biosystems, Waltham, MA) (8). All primers (*SLC16A3*, *TNFα*, *IL-6*, *SERPINE1*, *NOX-1*, *CLDN2*, *MUC2*, and *ZO-1* (*TJP1*)) were acquired from Bio-Rad (Hercules, CA, USA). The relative mRNA expression of each gene was expressed as a fold-change using the equation 2^−ΔΔCt^. Each PCR reaction was performed in triplicates.

### 2.3 Measurement of lactate levels in THP-1 supernatant

Phorbol 12-myristate-13-acetate (PMA)-activated THP-1 cells were plated in 100 µL of 5 × 10^5^ cells/mL in 96-well opaque-sided plates for 48 h. Dialyzed FBS (Thermo Fisher Scientific, Waltham, MA, USA), which contains significant reductions in supplemented lactate and small molecules, was used as a substitute for regular FBS in THP-1 media for this assay. The supernatant was collected 24 h after MAP infection, and the control wells were left uninfected. We used the Lactate-Glo™ luminescence assay from Promega™ (Madison, WI, USA) to measure the lactate levels in the collected supernatants, following the manufacturer’s instructions. Luminescence was recorded using the Promega™ GloMax Navigator system GM-2000 (Madison, WI, USA) and is reported in Relative Luminescence Units (R.L.U).

### 2.4 Inhibition of TLR2 in THP-1 macrophages

Differentiated THP-1 cells were treated with 200 µM of the small-molecule inhibitor of TLR2, TL2-c29 (InvivoGen, San Diego, CA, USA), for 3 h before being infected with MAP. The supernatant or RNA was collected from treated and control THP-1 cells 24 h after MAP infection for further downstream experiments. TNF-α and IL-6 expression was used to assess the efficiency of TLR2 inhibition during MAP infection.

### 2.5 Measurement of TNFα, IL-6, and sIL-6R released by THP-1

The supernatants of the THP-1 control cells and the intervention groups were collected 24 h after MAP infection. ELISA assays for TNF-α, IL-6, and sIL-6R (Thermo Fisher Scientific, Waltham, MA, USA) were performed according to the manufacturer’s instructions. Absorbance was read at 450 nm using a Multiskan FC plate reader (Thermo Fisher Scientific, Waltham, MA, USA). All treatment groups were tested in three technical repeats.

### 2.6 Inhibition of MCT4 using α-Cyano-4-hydroxycinnamic acid in THP-1 cells

CHCα is a non-specific inhibitor of monocarboxylate transporters including MCT4 (19). CHCα was acquired from MilliporeSigma (Burlington, MA, USA). A stock solution was prepared at 132.15 mM in methanol. Differentiated THP-1 cells were treated with 0.25 mM CHCα for 3 h before infection with MAP. Approximately 24 h later, the supernatant and RNA were harvested from cell culture wells for gene expression. Control cells were left untreated, uninfected, or infected with MAP without prior treatment with CHCα.

Supernatants of the differently treated THP-1 cells were also used to replace the supernatant of cultured HT-29 cells for 24 h. Intestinal cells were lysed, and RNA was used for qRT-PCR to evaluate the expression of *NOX-1* and *MUC2*.

### 2.7 Co-culturing THP-1 and Caco-2

THP-1 cells were grown and differentiated with PMA on 12-well plate inserts with a pore size of 0.3 µm at 1 mL of 5 × 10^5^ cells/mL (Greiner Bio-One, Kremsmünster, Austria), whereas Caco-2 cells were plated in 12-well plates at 2 ml of 3.5 × 10^5^ cells/mL. Following the differentiation of each cell type, THP-1 inserts were placed on top of Caco-2 wells. Both cell lines were treated with 0.25 mM CHCα for 3 h, and THP-1 cells were infected with MAP for 24 h. Caco-2 cells were lysed and gene expression was analyzed. Control cells were either left untreated and uninfected or infected with MAP without prior treatment with CHCα.

### 2.8 Statistical analysis

We used a two-tailed nonparametric sample t-test for statistical significance analysis to compare the groups in question. All analyses were performed using GraphPad Prism V.7.02 software (GraphPad, La Jolla, CA, USA). All experiments were performed in biological triplicates or more. Results are expressed as the mean ± SD. *Indicates P-values less than 0.05, **indicates P-values less than 0.005, and ***indicates P-values less than 0.001.

## 3 Results

### 3.1 MAP infection upregulates MCT4 expression and lactate export in THP-1 macrophages

**Figure 1:**
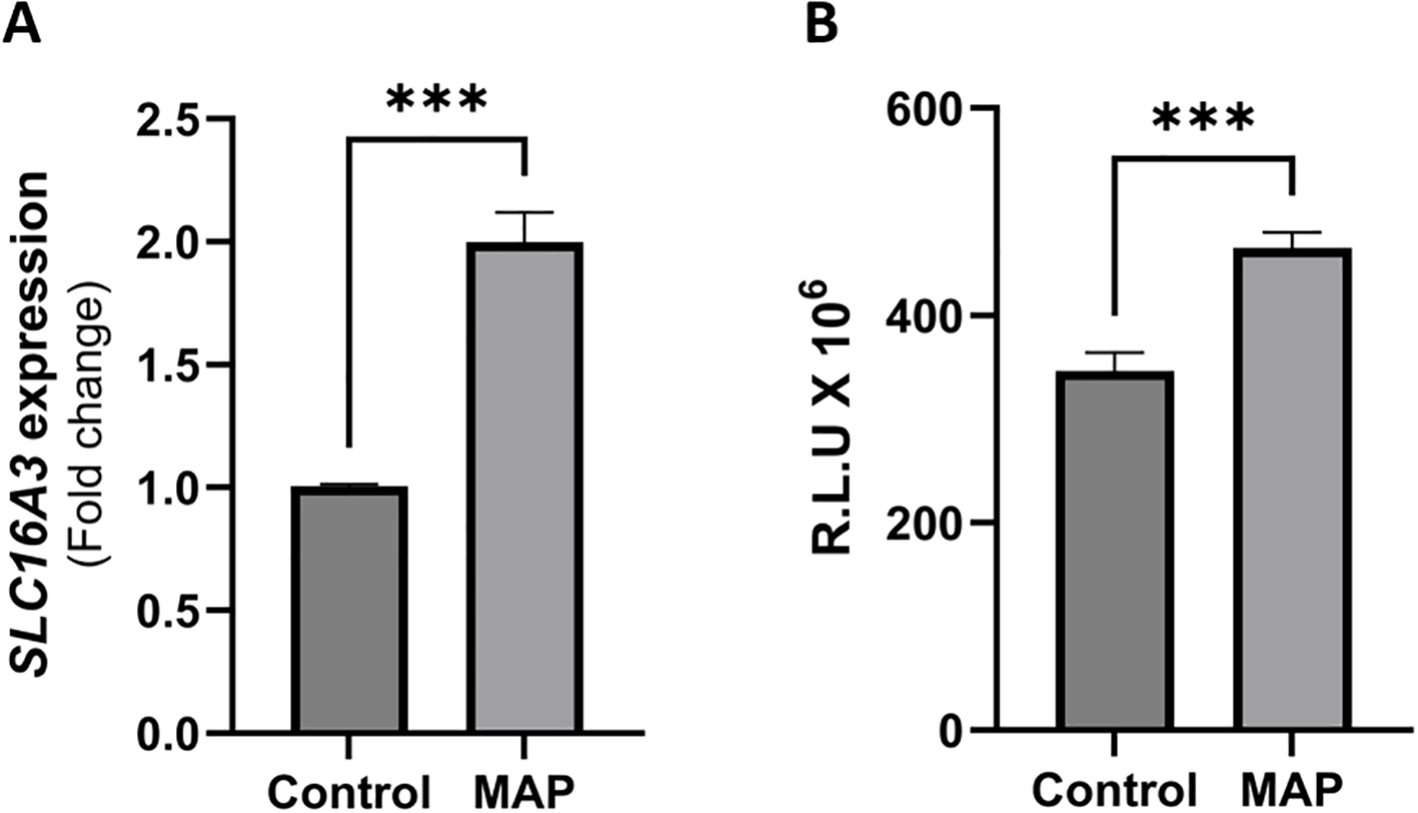
MAP infection upregulates MCT4 expression and lactate export in THP-1 macrophages. **(A)** THP-1 cells differentiated with PMA for 48 h were infected with 1 × 10^7^ CFU/mL MAP strain UCF4 for 24 h, followed by qRT-PCR for MCT4 (*SLC16A3*) expression (n = 3). **(B)** THP-1 cells were differentiated and plated in dialyzed FBS medium, followed by MAP infection. Lactate present in the supernatant was measured using a luminescence assay, and the results are expressed in Relative Luminescence Units (R.L.U) (n = 3). ***indicates P-values less than 0.001.

Metabolic switching to aerobic glycolysis has been reported in macrophages activated by intracellular pathogens, such as *Mtb* (20). To determine the relationship between MAP infection and MCT4 activity reflective of glycolysis status, we analyzed the RNA expression of MCT4 gene, *SLC16A3*, and measured the lactate levels released by THP-1 cells following MAP infection. Our results showed a doubling of *SLC16A3* expression (2 ± 0.12-fold, P-value <0.001) in MAP infection compared to uninfected THP-1 cells (**Figure 1A**). This enhanced expression is validated functionally by an upregulation in exported lactate by MAP-infected THP-1 compared to uninfected cells (465.3 ± 14.97 R.L.U vs. 346.9 ± 17.38 R.L.U, respectively, P-value <0.001) as presented in **Figure 1B**. These findings indicate increased glycolysis during MAP infection in macrophages and a specific reliance on MCT4.

### 3.2 TLR2 is partially responsible for MAP-mediated upregulation of MCT4 expression by THP-1 cells

**Figure 2:**
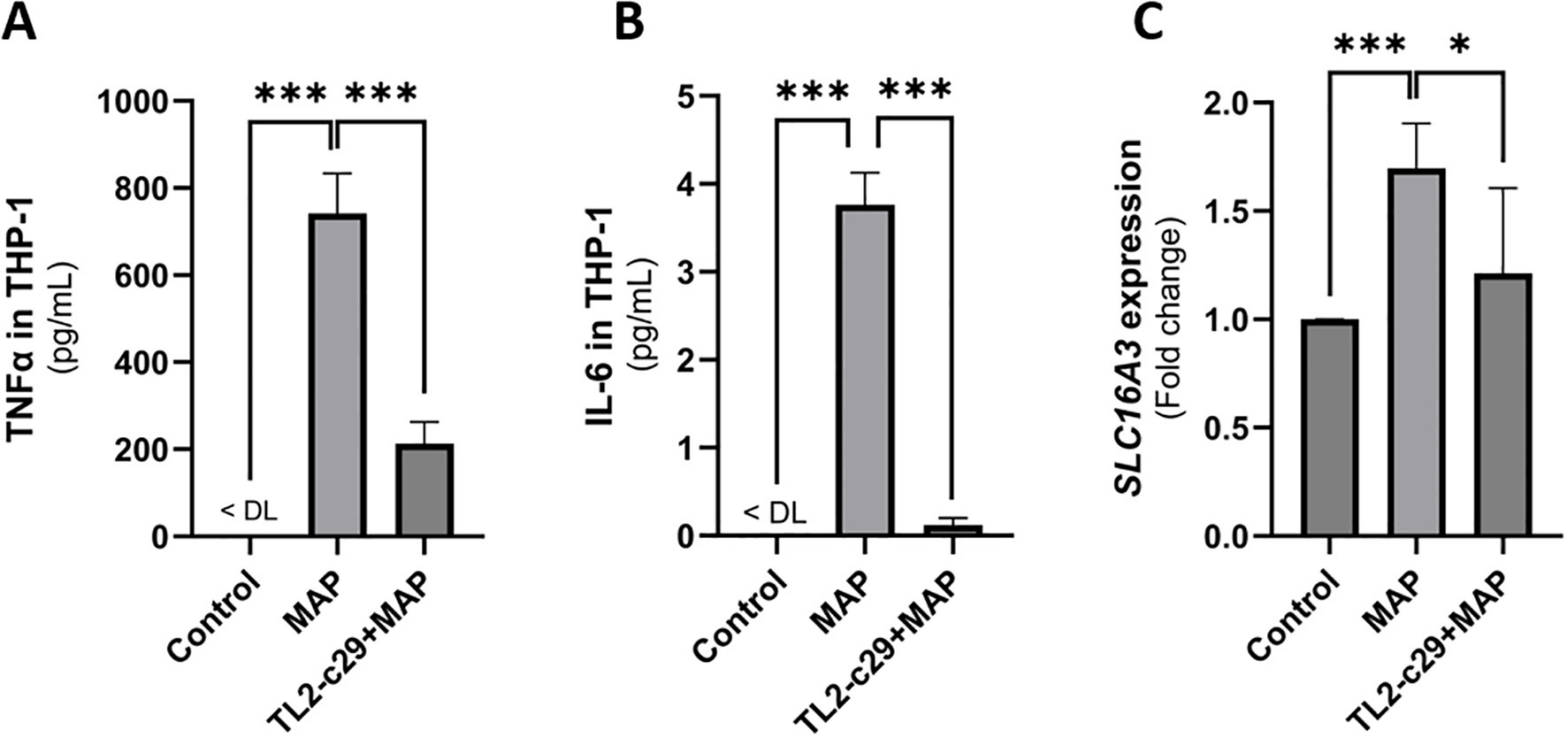
TLR2 is partially responsible for the MAP-mediated upregulation of MCT4 expression in THP-1 cells. THP-1 macrophages were treated with 200 µM TLR2 inhibitor TL2-c29 for 3 h before MAP infection. The supernatant was collected for ELISA determination of **(A)** TNF-α (n = 3) and **(B)** IL-6 (n = 3) concentrations to validate TLR2 inhibition. **(C)** MCT4 (*SLC16A3*) expression was measured in these cells by qRT-PCR (n = 6). The values below the detection limit of the ELISA kit were 0. DL, detection limits. *Indicates P-values less than 0.05, and ***indicates P-values less than 0.001.

Previous work by our lab has shown the importance of TLR2 activation by MAP in activating macrophage responses (21, 22). In this study, we investigated the role of TLR2 activation in MCT4 upregulation in MAP-infected THP-1 macrophages. TLR2 signaling was inhibited using a selective small-molecule inhibitor, TL2-c29 (200 µM). To validate TLR2 inhibition, we measured TNF-α and IL-6 levels with and without TL2-c29 treatment. **Figure 2A** shows the successful blocking of TLR2 activation by TL2-c29 during MAP infection, as seen from the drop in TNFα released by THP-1 cells from 742.4 ± 91.62 pg/mL (P-value <0.001) in MAP-infected cells to 213.1 ± 49.91 pg/mL (P-value <0.001) in cells pre-treated with TL2-c29. IL-6 levels also validate TLR2 inhibition as they decreased from 3.77 ± 0.36 pg/mL (P-value <0.001) in MAP-infected THP-1 cells to 0.12 ± 0.08 pg/mL (P-value <0.001) in pre-treated cells (**Figure 2B**). Following confirmation of TLR2 inhibition, we measured MCT4 (*SLC16A3*) expression in the same cells (**Figure 2C**). MAP upregulated *SLC16A3* by 1.7 ± 0.2 folds (P-value <0.001), while TLR2 inhibition reduced it to 1.21 ± 0.39-fold (P-value <0.05) compared to control cells. In conclusion, TLR2 activation by MAP is essential for MCT4 upregulation in infected macrophages.

### 3.3 Inhibiting MCT4 with CHCα reduces inflammatory cytokine release by THP-1 cells during MAP infection

**Figure 3:**
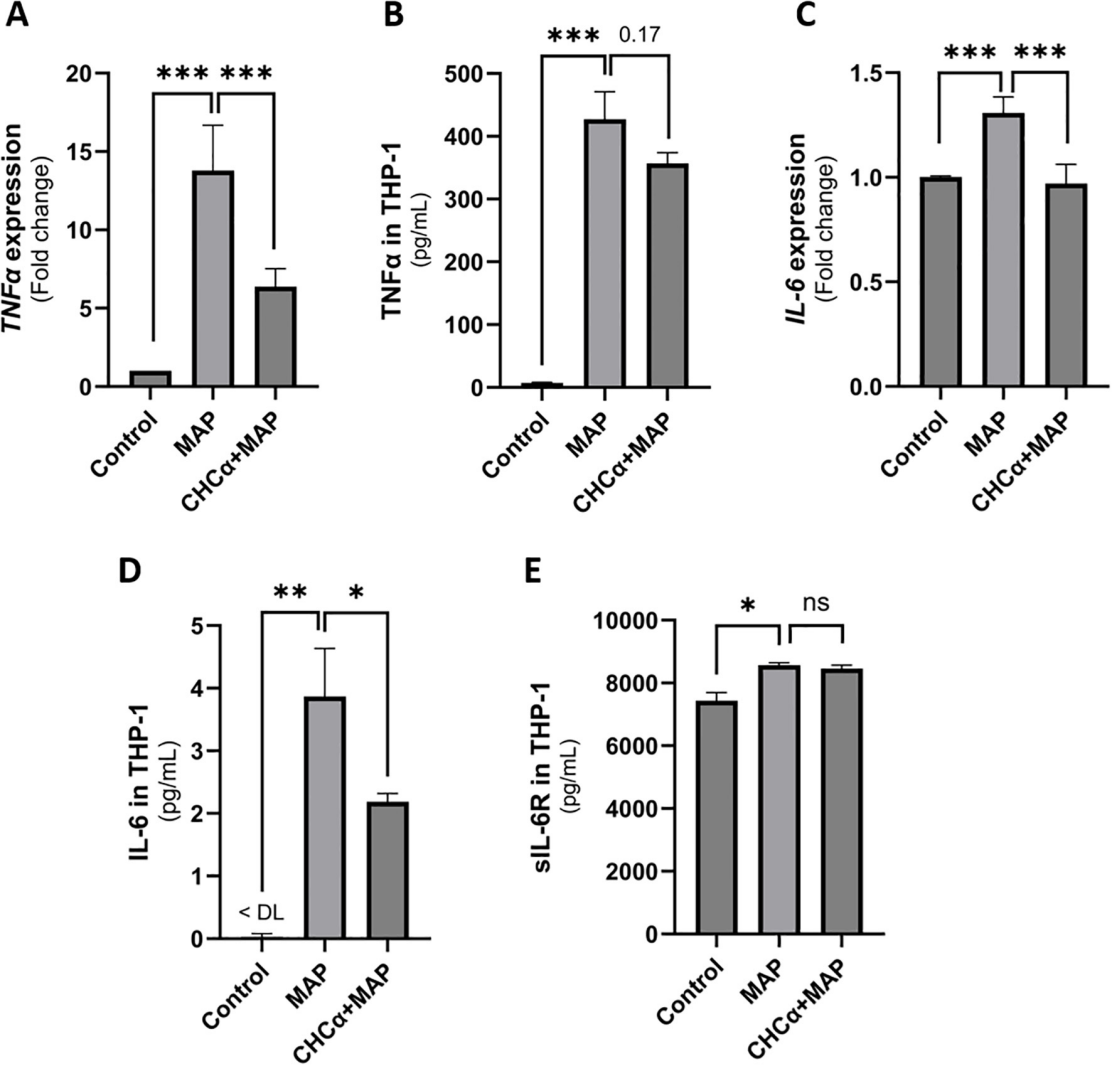
Inhibition of MCT4 with CHCα reduces inflammatory cytokine release by THP-1 cells during MAP infection. THP-1 cells were treated with 0.25 mM CHCα for 3 h before infection with MAP. RNA and supernatants were collected after 24 h. **(A)** qRT-PCR analysis of *TNFA* expression in THP-1 cells. **(B)** TNFα ELISA of THP-1 supernatant. **(C)** qRT-PCR expression of *IL-6* in THP-1. **(D)** IL-6 ELISA of THP-1 supernatant. **(E)** sIL-6R ELISA of the THP-1 supernatant. Values below the detection limits of the ELISA kit were assigned 0. DL, detection limits. ns, not significant. *Indicates P-values less than 0.05, **indicates P-values less than 0.005, and ***indicates P-values less than 0.001.

To evaluate the role of MCT4 activity in the inflammatory response of macrophages during MAP infection, we inhibited MCT4 expression in THP-1 cells using CHCα (as previously described (12)) before exposing them to MAP. In **Figure 3**, we see a reversal in the upregulation of the inflammatory cytokines TNF-α and IL-6 during MAP infection. As shown in **Figure 3A**, MAP upregulated *TNFA* expression to 13.79 ± 2.88 folds compared to control cells (P-value <0.001), and CHCα pre-treatment reduced *TNFA* expression to 6.37 ± 1.16 folds (P-value <0.001 compared to MAP-infected cells). This was further confirmed by ELISA performed on the supernatants collected from the cells. MAP infection upregulated TNF-α release by THP-1 cells in the supernatant to 427.4 ± 43.83 pg/mL (P-value <0.001) compared to 7.22 ± 1.1 pg/mL in control cells (**Figure 3B**). This is reduced to 356.7 ± 17.19 pg/mL (P-value = 0.17) with CHCα pre-treatment. IL-6 is also significantly reduced by CHCα treatment (0.97 ± 0.09 folds, P-value <0.001 and 2.19 ± 0.13 pg/mL, P-value <0.05) during MAP infection compared to untreated MAP-infected cells (1.31 ± 0.08 folds and 3.87 ± 0.77 pg/mL) as shown in **Figures 3C, D**. However, shedding of the IL-6 receptor (soluble IL-6 receptor; sIL-6R) during MAP infection was unaffected by MCT4 inhibition and CHCα treatment (**Figure 3E**). MAP upregulated the levels of sIL-6R to 8,574 ± 71.39 pg/mL (P-value <0.05) compared to control cells (7,437 ± 261.8 pg/mL), which did not differ from the CHCα-pretreated cells (8,462 ± 110.6 pg/mL). Overall, our findings prove the reduction of the inflammatory capacity of MAP-infected macrophages by MCT4 inhibition.

### 3.4 Inhibition of MCT4 in THP-1 during MAP infection restores basal intestinal cell NOX-1 and MUC2 expression

**Figure 4:**
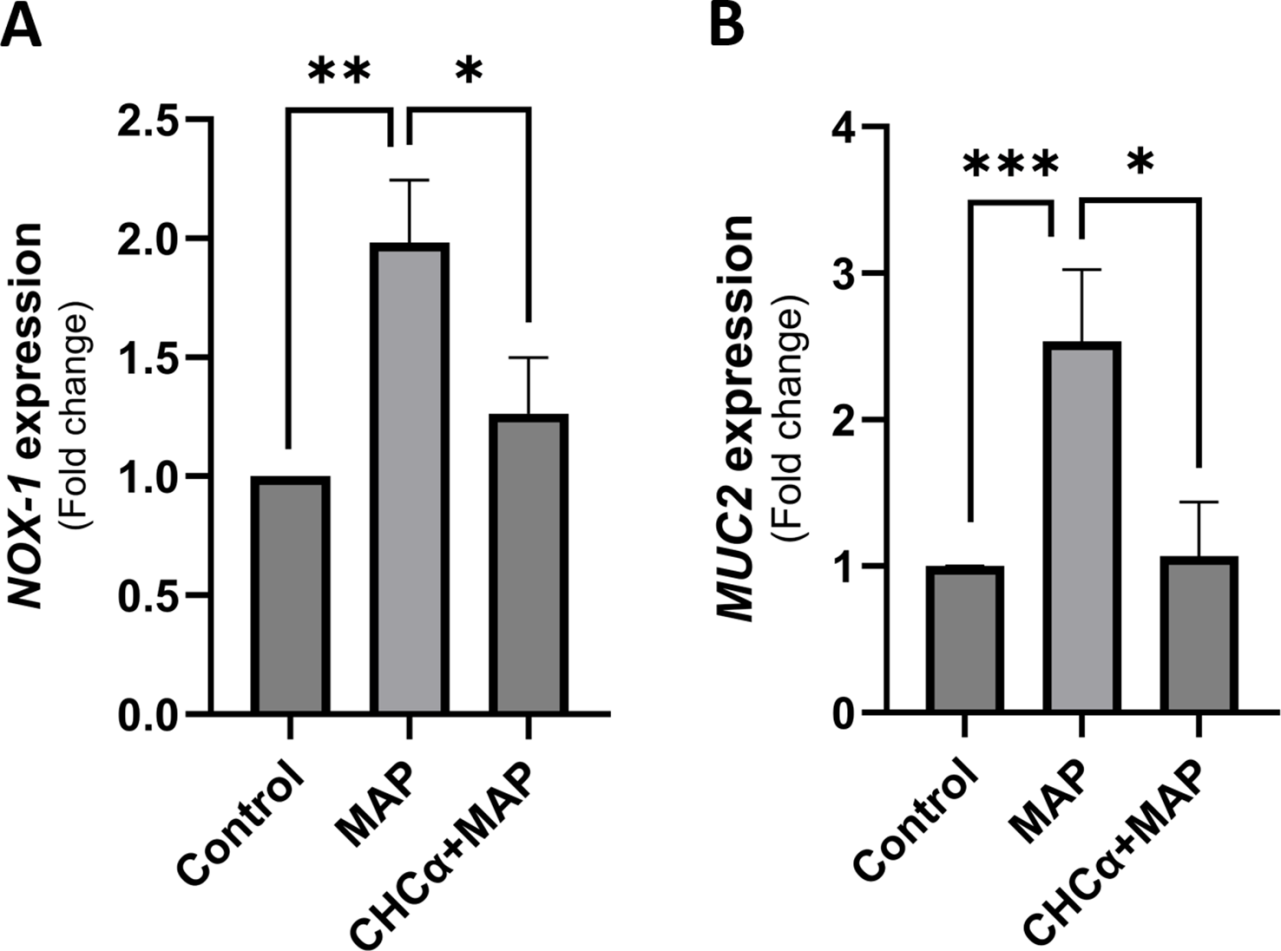
Inhibition of MCT4 in THP-1 cells during MAP infection restores basal intestinal cell oxidative status and mucin production. The supernatants of THP-1 cells under different conditions of CHCα treatment and MAP infection were collected 24 h after infection. These were used to substitute the culture media for growing and differentiated HT-29 cells for 24 h before RNA extraction. **(A)** qRT-PCR analysis of *NOX-1* expression in HT-29 cells (n = 3). **(B)** qRT-PCR analysis of *MUC2* expression in HT-29 cells (n = 5). *Indicates P-values less than 0.05, **indicates P-values less than 0.005, and ***indicates P-values less than 0.001.

To reflect the consequences of MCT4 inhibition in MAP-infected macrophages on intestinal epithelial health, we treated cultured HT-29 cells with the conditioned media of THP-1 cells treated with CHCα and infected with MAP, as previously mentioned. We measured the expression of NOX-1 and MUC2, which indicate oxidative stress and mucin production by the intestinal cells, respectively. As shown in **Figure 4A**, *NOX1* was upregulated during MAP infection to 1.98 ± 0.26 folds (P-value <0.005) and reduced to 1.26 ± 0.24 folds (P-value <0.05, compared to control cells) with CHCα treatment. Mucin expression was restored to normal levels (**Figure 4B**). MAP infection upregulated *MUC2* expression to 2.53 ± 0.49-fold (P-value <0.001), and CHCα treatment restored production levels to baseline (1.07 ± 0.37-fold, P-value <0.05 compared to MAP infection conditions). These findings highlight the effects of MCT4 inhibition in MAP-infected THP-1 cells in restoring the baseline intestinal epithelial conditions.

### 3.5 Dual inhibition of MCT4 in THP-1 and Caco-2 during MAP infection reduces inflammatory proteins released by intestinal cells

**Figure 5:**
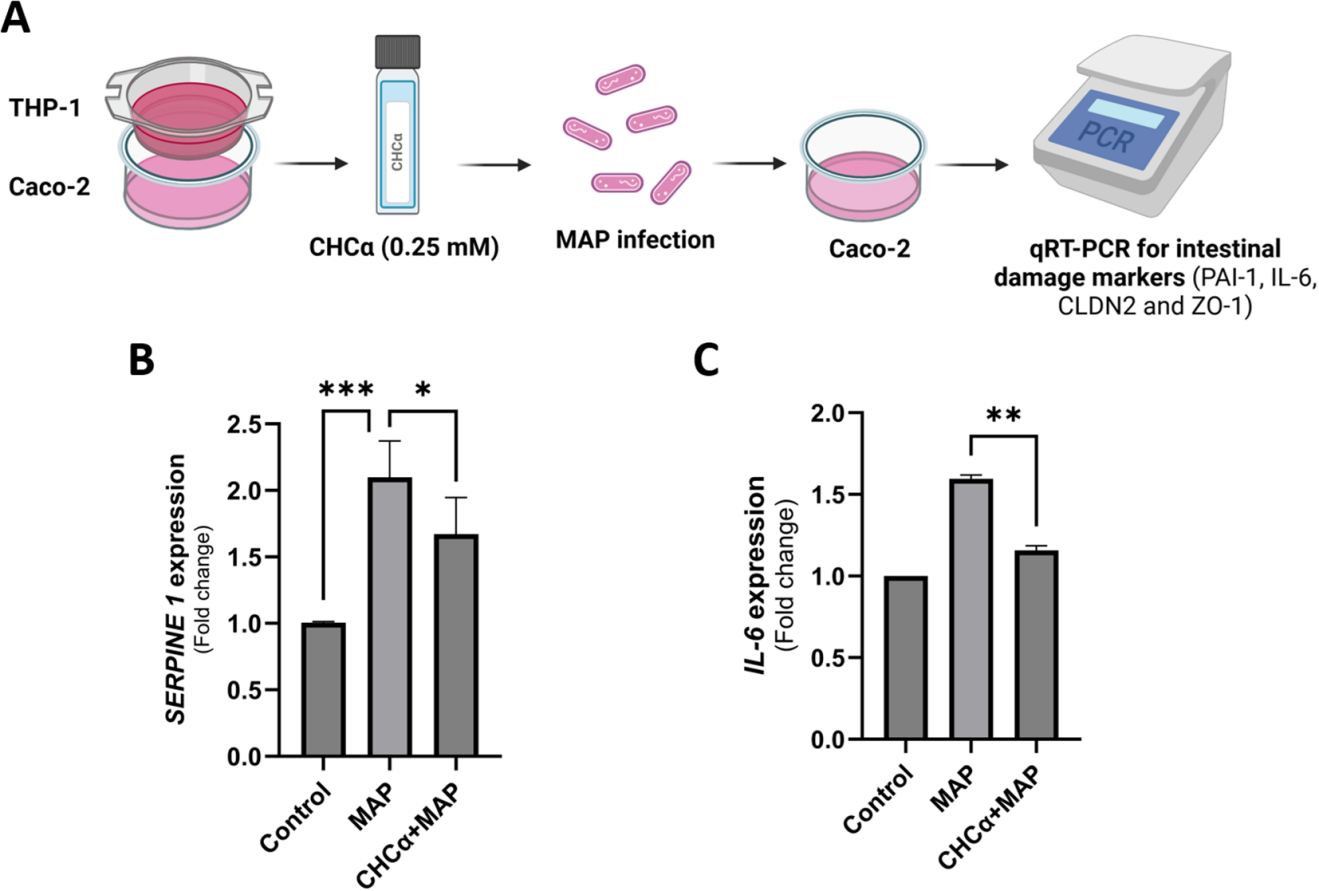
Dual inhibition of MCT4 in THP-1 and Caco-2 cells during MAP infection reduced the release of inflammatory proteins by intestinal cells. **(A)** THP-1 and Caco-2 cells were co-cultured in inserts and wells, respectively. Both cell lines were treated with 0.25 mM CHCα for 3 h, and then THP-1 macrophages were infected with MAP for 24 h. Caco-2 cells were harvested for qRT-PCR analysis of the target genes. **(B)** qRT-PCR analysis of *SERPINE1* expression in Caco-2 cells (n = 6). **(C)** qRT-PCR analysis of *IL-6* expression in Caco-2 cells (n = 3). *Indicates P-values less than 0.05, **indicates P-values less than 0.005, and ***indicates P-values less than 0.001.

To comprehensively investigate the role of MCT4 inhibition during MAP infection, we co-cultured THP-1 and Caco-2 cells in an insert-well system. We treated the cells simultaneously with CHCα before MAP infection (**Figure 5A**). We then measured the expression of colitis-aggravating factor, SERPINE1/PAI-1, and endogenous IL-6 levels in Caco-2 cells. As shown in **Figure 5B**, MAP upregulated *SERPINE1* expression by 2.1 ± 0.27 folds (P-value <0.001) compared to control cells. CHCα treatment reduced the upregulation of the inflammatory marker to 1.67 ± 0.28 folds (P-value <0.05, compared to MAP infection conditions). *IL-6* was also upregulated by MAP infection in Caco-2 cells to 1.6 ± 0.02 folds (P-value <0.001), as seen in **Figure 5C**, and this was reversed to normal levels by CHCα pre-treatment (1.16 ± 0.03 folds, P-value <0.005). In conclusion, MCT4 inhibition is showing promising effects in attenuating the inflammatory phenotype of intestinal cells during MAP infection.

### 3.6 Dual inhibition of MCT4 in THP-1 and Caco-2 during MAP infection upregulates the expression of *TJP1* (*ZO-1)* but not of *CLDN2*

**Figure 6:**
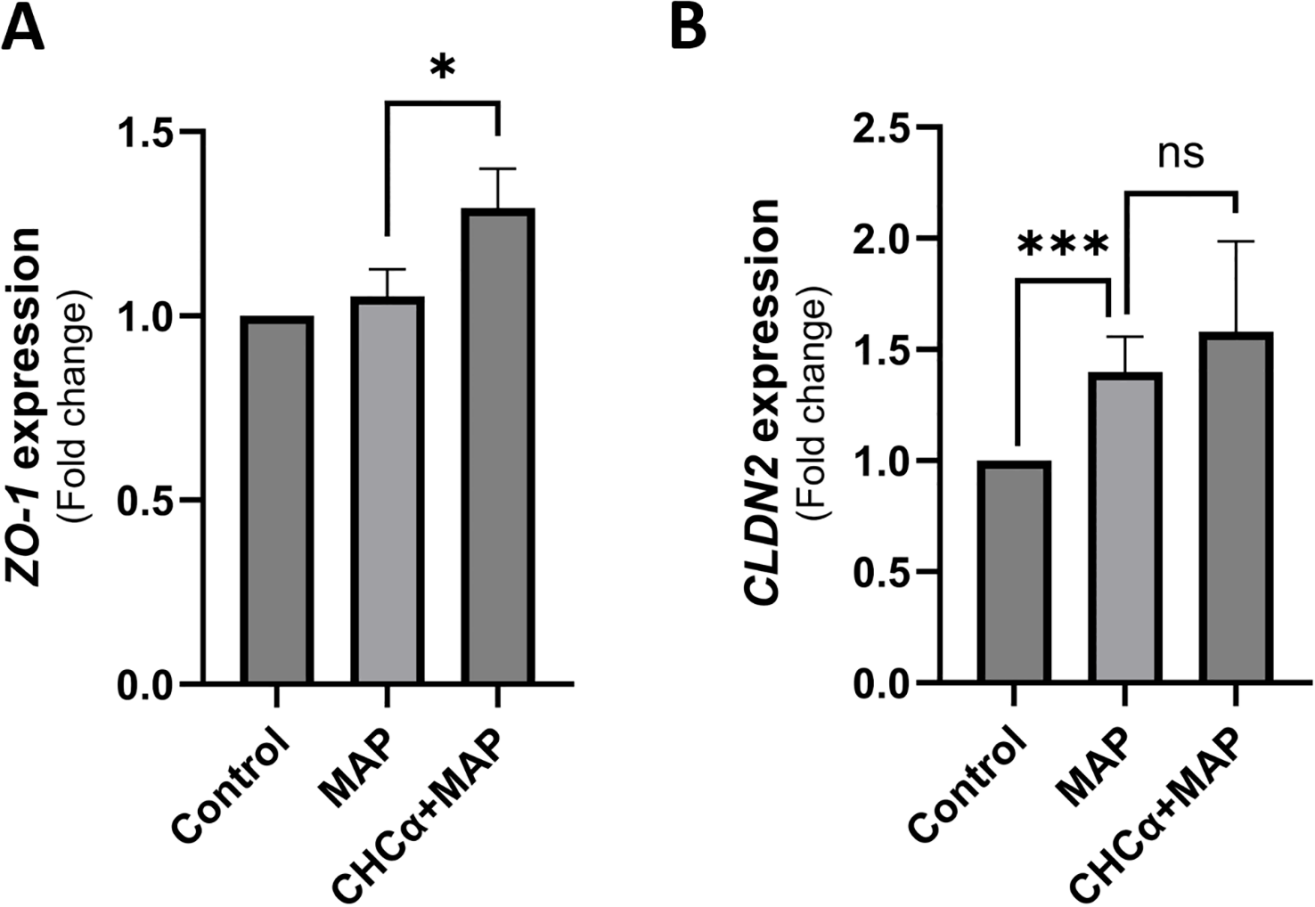
Dual inhibition of MCT4 in THP-1 and Caco-2 cells during MAP infection upregulates the expression of *TJP1* (ZO-1) but not *CLDN2*. THP-1 and Caco-2 cells were co-cultured in inserts and wells, respectively. Both cell lines were treated with 0.25 mM CHCα for 3 h, and then THP-1 cells were infected with MAP for 24 h. **(A)** qRT-PCR analysis of *TJP1*/*ZO-1* expression in Caco-2 cells (n = 3). **(B)** qRT-PCR of *CLDN2* expression in Caco-2 cells (n = 6). *Indicates P-values less than 0.05, and ***indicates P-values less than 0.001. ns, not significant.

Following up on the assessment of MCT4 inhibition during MAP infection on intestinal epithelial homeostasis, we measured changes in the expression of key tight junction proteins involved in IBDs, Zonula Occludens-1/Tight junction protein-1 (ZO-1/TJP1) and claudin-2 (CLDN2) (23). **Figure 6A** shows that MAP infection does not affect *TJP1* expression in Caco-2 cells (1.05 ± 0.07 folds, P-value = 0.28) compared to control cells. Interestingly, MCT4 inhibition via CHCα treatment upregulated the expression of *TJP1* by 1.29 ± 0.11 folds (P-value <0.05). On the other hand, *CLDN2* is upregulated by MAP infection to 1.4 ± 0.16 folds (P-value <0.001), and pre-treatment with CHCα had no significant effect on reversing that (1.58 ± 0.41 folds, P-value = 0.38) as shown in **Figure 6B**. Overall, we showed an upregulatory effect of MCT4 inhibition on *ZO-1* expression during MAP infection.

## 4 Discussion

MCT4 is an emerging drug target for the treatment of several cancers; however, it is yet to be considered for the treatment of IBDs (24–27). Here, we highlight the role of MCT4 in MAP-associated CD pathogenesis, focusing on the inflammatory response of macrophages and their subsequent effects on the integrity and function of intestinal epithelial cells. Specifically, we demonstrated that MAP infection drives a metabolic shift in THP-1 macrophages to aerobic glycolysis through upregulation of MCT4 expression and lactate export observed in MAP-infected cells. This phenotype is primarily dependent on the activation of TLR2 signaling in THP-1 macrophages. This metabolic shift allows the inflammatory polarization of these macrophages and the production of the inflammatory cytokines TNFα and IL-6. Inhibition of MCT4 in macrophages reversed the inflammatory phenotype and MAP-imposed changes NOX-1 and MUC2 expression in intestinal cells. Ultimately, the dual inhibition of MCT4 in both macrophages and intestinal cells reduced the inflammatory characteristics of intestinal cells and modulated the expression of tight junction proteins.

The upregulation of MCT4 (*SLC16A3*) expression and lactate export by MAP-infected THP-1 macrophages (**Figure 1**) indicates a glycolytic shift, which is supported by previous studies on different intracellular microbial strains, including *Mtb* (28–30). This is also supported by a study showing upregulation of hypoxia-inducible factor 1α (HIF1α) in hypoxic or microbe-stimulated macrophages, which activates the expression of glycolytic enzymes and assists in the shift of macrophages to their inflammatory state (M1) (31). Aerobic glycolysis usually (1) provides metabolic intermediates needed for cell growth and differentiation, (2) allows faster energy production to cope with the inflammatory state of the cell, and (3) provides biosynthetic intermediates that support host defenses against microbes (32–34).

MAP activates the inflammatory response in macrophages by activating TLR2 and MYD88 signaling pathways, which eventually leads to NF-κB activation (35, 36). We showed that TLR2 activation initiates the signaling cascade within macrophages, leading to MCT4 upregulation during MAP infection. This is in agreement with previous studies showing a direct relationship between TLR2 activation and MCT4 upregulation in an MYD88-dependent pathway. However, the partial reduction in MCT4 expression after TLR2 inhibition suggests the involvement of other pathways (possibly TLR4) in regulating MCT4 expression (12).

The upregulation of inflammatory cytokines TNF-α and IL-6 in MAP-infected THP-1 cells is counteracted by MCT4 inhibition, which may lead to glycolytic acidosis due to intracellular lactate accumulation (11). These findings support the role of MCT4 in facilitating the inflammatory activation of macrophages during MAP infection by mediating the efflux of intracellular lactate generated by the enhanced glycolytic pathway. Silencing MCT4 in cultured macrophages reduces the levels of TNF-α and IL-6 released by lipopolysaccharide (LPS) stimulation (12). Interestingly, MCT4 inhibition did not affect the shedding of IL-6R, which contributes to the trans-signaling activity of IL-6. This shows selectivity for cytokine expression but not metalloproteinase (specifically, ADAM17, a disintegrin and metalloproteinase) activity, which is responsible for IL-6R cleavage and shedding from the surface of macrophages (37). The reduced inflammatory phenotype of macrophages treated with CHCα restored the intestinal markers (*NOX-1* and *MUC2*) to baseline levels in HT-29 cells incubated with conditioned media. MUC2, which is the predominant mucin that formulates the mucous layer of the intestine and isolates luminal bacteria and antigens from the epithelial cells and underlying stroma, was restored to normal levels, indicating a reversal of the signaling pathways activated by MAP infection (38, 39). These could potentially involve NF-κB and/or CREB, which can bind to the MUC2 promoter and enhance its activity (40). Increased MUC2 expression in intestinal cells leads to the accumulation of misfolded MUC2 in the endoplasmic reticulum (ER) and the production of reactive oxygen species (ROS) by mitochondria. This leaves goblet cells, which are the MUC2-producing intestinal cells, more susceptible to ER stress and consequent death by apoptosis (41). Restoring MUC2 expression to baseline under MAP infection should alleviate ER stress and intestinal epithelial barrier dysfunction. NOX-1 (NADPH oxidase-1), a marker of oxidative stress in intestinal cells, was indeed restored to baseline levels. These findings highlight the role of MCT4 inhibition in reversing the effects of MAP infection on intestinal epithelial cell function, by alleviating the inflammatory characteristics of macrophages.

Our co-culture system consisting of THP-1 macrophages and Caco-2 intestinal cells revealed that the dual inhibition of MCT4 in both cell types reduced colitis-related inflammatory markers such as SERPINE1/PAI-1 and IL-6 in intestinal cells. MCT4 overexpression in intestinal cells has previously been linked to IL-6 upregulation. MCT4 leads to the phosphorylation and nuclear translocation of the p65 subunit of NF-κB, which binds to the IL-6 promoter to induce gene expression in Caco-2 cells (14). Serpin Peptidase Inhibitor 1 (SERPINE1), which encodes for Plasminogen Activator Inhibitor-1 (PAI-1), inhibits fibrinolysis (42). PAI-1 exacerbates mucosal damage in colitis models by inhibiting the activation of the anti-inflammatory cytokine TGFβ, and PAI-1 inhibition alleviates intestinal inflammation (43). SERPINE1/PAI-1 is highly expressed in the mucosa and serum of untreated IBD patients with active disease. SERPINE1/PAI-1 expression correlates positively with disease activity and can be used to monitor therapeutic responses (44). SERPINE1 is thus an essential marker of intestinal inflammation and mucosal damage. The inflammatory effect of MCT4 on intestinal cells has been demonstrated by the upregulation of intestinal NLRP3 inflammasome activity due to MCT4 ectopic expression. This leads to increased cell pyroptosis and the release of the inflammatory cytokines IL-1β and IL-18, which aggravate intestinal inflammation (15). Our findings emphasize the role of MCT4 expressed by intestinal cells and macrophages in inflammatory damage of the intestines during MAP infection.

Interestingly, MCT4 inhibition during MAP infection had different effects on tight junction protein expression. MAP infection upregulates the leaky tight junction protein CLDN2, but does not affect ZO-1 (8). Similarly, CLDN2 is upregulated in cultured intestinal cell lines (HT-29 and T84) by other microbial products, such as cholera toxin and staphylococcal enterotoxin B (45). CLDN2 increases barrier permeability and facilitates the internalization of protein antigens across the epithelial monolayer, contributing to intestinal inflammation and barrier dysfunction (45). The dual inhibition of MCT4 using CHCα failed to reverse the upregulation of CLDN2 during MAP infection, but successfully elevated ZO-1. These findings indicate that the pathway stemming from MCT4 inhibition is selective for ZO-1 but not CLDN2 expression. Although ZO-1 expression is usually reduced in patients with IBD, we found no difference in our *in vitro* MAP infection model (46). ZO-1 is vital for intestinal cell proliferation, specifically in response to injury, through mitotic spindle regulation and Wnt signaling, the latter of which controls cell fate and tissue development (46). The upregulation of ZO-1 in our study could indicate improved mucosal repair capabilities with MCT4 inhibition during MAP infection; however, confirmation in an *in vivo* model is needed. MCT4 overexpression experiments in Caco-2 cells also showed a reduction in ZO-1 gene expression due to reduced promoter activity and a consequent decrease in transepithelial electrical resistance (TEER) value supporting our findings of increased ZO-1 expression following MCT4 inhibition, which is upregulated during MAP infection (14). A technical disadvantage of our co-culture model was the inability to perform a TEER assay because Caco-2 cells were grown in a well, whereas THP-1 cells were cultured in an insert on top, and for TEER performance Caco-2 would need to be grown in the insert. Measuring TEER would provide important insights into barrier function and permeability.

Monocarboxylate transporters (MCTs), which include four functional members in humans (MCT1, MCT2, MCT3, and MCT4), mediate the proton-coupled transport of monocarboxylic acids, such as pyruvate, lactate, and ketone bodies, across cellular membranes (47). The role of other MCTs should be considered, and a more specific inhibition of MCT4, such as siRNA or the clinical candidate inhibitor AZD0095, should be tested to further validate our findings regarding the role of MCT4 in MAP-associated pathogenesis (16).

In the absence of a viable *in vivo* model for CD associated with MAP infection, this study, by far, has provided significant insights into tracking and characterizing MAP infection in CD-like macrophages and its impact on intestinal integrity and function. It also provides a foundation for the evaluation of MCT4 inhibition and its therapeutic potential in animal and clinical settings. This study provides an alternative for the effective treatment of CD, especially in cases associated with MAP infection. Our group recently reported that IL-6 blockade during MAP infection leads to exacerbated epithelial damage (8). Likewise, TNFα inhibition during MAP infection allows the bacterium to persist in macrophages by neutralizing host anti-microbial responses (7). However, this study supports a better therapeutic option using MCT4 inhibitors in CD patients with underlying MAP infection.

## Data Availability

The original contributions presented in the study are included in the article/supplementary material. Further inquiries can be directed to the corresponding author.
