# Corrigendum: MCT4 inhibition attenuates inflammatory response to *Mycobacterium avium paratuberculosis* infection and restores intestinal epithelial integrity *in vitro*

**DOI:** 10.3389/fimmu.2025.1613909

**Published:** 2025-05-14

**Authors:** Ala’ Alhendi, Saleh A. Naser

**Affiliations:** Division of Molecular Microbiology, Burnett School of Biomedical Sciences, College of Medicine, University of Central Florida, Orlando, FL, United States

**Keywords:** MCT4, *Mycobacterium avium paratuberculosis* (MAP), TLR-2, Crohn’s disease (CD), SERPINE1, tight junction

In the published article, there was an error in **Figure 4** as published. **Figure 4** as it appears in the published article is the same as Figure 5 by mistake (specifically panel B and C of Figure 5). The corrected **Figure 4** and its caption appear below.

**Figure 4:**
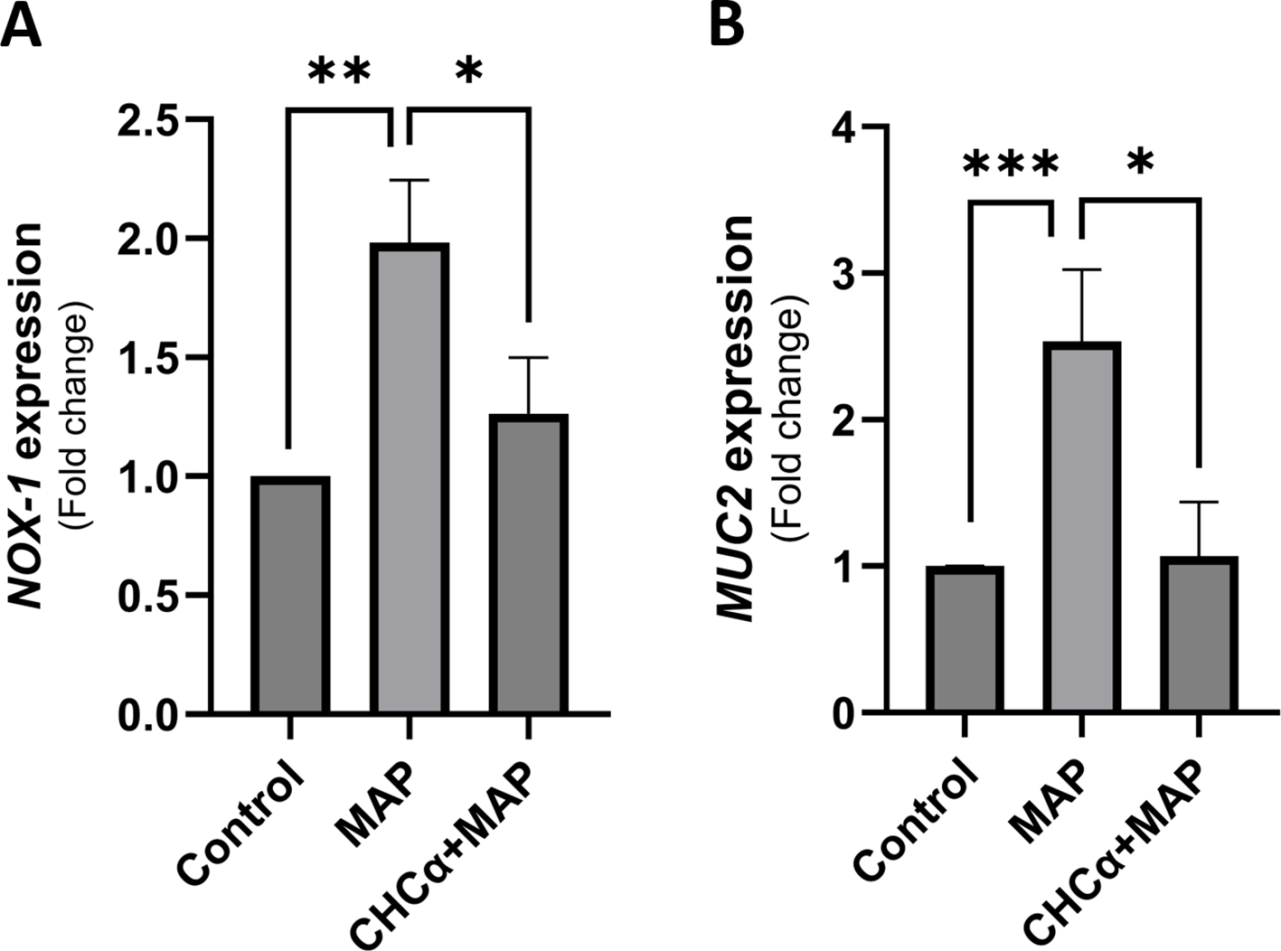
Inhibition of MCT4 in THP-1 cells during MAP infection restores basal intestinal cell oxidative status and mucin production. The supernatants of THP-1 cells under different conditions of CHCα treatment and MAP infection were collected 24 h after infection. These were used to substitute the culture media for growing and differentiated HT-29 cells for 24 h before RNA extraction. **(A)** qRT-PCR analysis of *NOX-1* expression in HT-29 cells (n = 3). **(B)** qRT-PCR analysis of *MUC2* expression in HT-29 cells (n = 5). *Indicates P-values less than 0.05, **indicates P-values less than 0.005, and ***indicates P-values less than 0.001.

The authors apologize for this error and state that this does not change the scientific conclusions of the article in any way. The original article has been updated.

